# C-reactive protein to lymphocyte ratio is a significant predictive factor for poor short-term clinical outcomes of SARS-CoV-2 BA.2.2 patients

**DOI:** 10.3389/fpubh.2023.1168375

**Published:** 2023-04-06

**Authors:** Benjie Xiao, Yinyan Wu, Huazheng Liang, Jingjing Xiao, Yudi Han, Zhangwei Yang, Yong Bi

**Affiliations:** ^1^Department of Neurology, Shanghai Fourth People's Hospital, School of Medicine, Tongji University, Shanghai, China; ^2^Clinical Research Center for Anesthesiology and Perioperative Medicine, Shanghai Fourth People’s Hospital, School of Medicine, Tongji University, Shanghai, China; ^3^Monash Suzhou Research Institute, Suzhou, Jiangsu Province, China; ^4^Department of Information, Shanghai Fourth People's Hospital, School of Medicine, Tongji University, Shanghai, China; ^5^Shanghai University of Medicine & Health Sciences Affiliated Zhoupu hospital, Shanghai, China; ^6^Translational Research Institute of Brain and Brain-Like Intelligence, Shanghai Fourth People’s Hospital, School of Medicine, Tongji University, Shanghai, China

**Keywords:** SARS-CoV-2 BA.2.2, C-reactive protein to lymphocyte ratio, cut-off value, clinical outcomes, multivariate logistic regression

## Abstract

**Objective:**

The aim of the present study is to assess the utility of C-reactive protein to Lymphocyte Ratio (CLR) in predicting short-term clinical outcomes of patients infected by SARS-CoV-2 BA.2.2.

**Methods:**

This retrospective study was performed on 1,219 patients with laboratory-confirmed SARS-CoV-2 BA.2.2 to determine the association of CLR with short-term clinical outcomes. Independent Chi square test, Rank sum test, and binary logistic regression analysis were performed to calculate mean differences and adjusted odds ratios (aORs) with their 95% CI, respectively.

**Results:**

Over 8% of patients admitted due to SARS-CoV-2 BA.2.2. were critically ill. The best cut-off value of CLR was 21.25 in the ROC with a sensitivity of 72.3% and a specificity of 86%. After adjusting age, gender, and comorbidities, binary logistic regression analysis showed that elevated CLR was an independent risk factor for poor short-term clinical outcomes of COVID-19 patients.

**Conclusion:**

C-reactive protein to Lymphocyte Ratio is a significant predictive factor for poor short-term clinical outcomes of SARS-CoV-2 BA.2.2 inflicted patients.

## 1. Introduction

The 2019 corona virus disease (COVID-19) has elicited global chaos, whereas a novel Omicron variant has challenged the healthcare system (1). This variant has been referred to as a variant of concern (VOC) by the World Health Organization (WHO), owing to its alarming transmission and infectivity rate (2, 3). Currently, 26 countries are infected by Omicron variants (3). In late February, 2022, a wave of severe acute respiratory syndrome coronavirus-2 (SARS-CoV-2) infection rapidly appeared in Shanghai, China. Genomic analysis showed that the SARS-CoV-2 BA.2.2 sub-lineage was the responsible pathogen (4). Of note, BA.2 is a sub-lineage of the Omicron variant of SARS-CoV-2 (B.1.1.529). Although Omicron BA.2 evolves toward less virulence, a large percentage of patients with severe conditions have been reported in the unvaccinated population, especially in elderlies (5). How to translate the knowledge on COVID-19 into prevention and therapeutic strategies for Omicron variants is a problem we need to face now and in the future. In this retrospective study, we set to explore the potential of early triage using results of routine tests.

It is essential to early assess and classify disease severity in order to improve patients’ clinical outcomes. At the same time, classification can also help clinicians find patients who may have aggravated conditions as soon as possible. It is more beneficial to allocate limited medical resources to people who need more active treatments. Therefore, clinicians need more valuable laboratory indicators that can help to assess disease severity at the early stage of infection. One of the characters of COVID-19 is the systemic inflammatory response to the SARS-CoV-2 infection. Nearly all patients admitted to the hospital due to COVID-19 have anomalies in these inflammatory biomarkers (6). Recent studies have demonstrated an association between elevated levels of CRP and the severity of COVID-19 (7–11). Some studies even showed that the level of CRP was correlated with poor clinical outcomes among COVID-19 patients (6, 8, 12). Interestingly, some patients, particularly severe COVID-19 patients, showed a low lymphocyte (LYM) count in the full blood count (13–15). Studies have suggested that the degree of lymphocyte count reduction correlates with disease severity in patients with COVID-19 (14, 16, 17). It has been proposed that the ratio of CRP to lymphocytes is the best predictor of survival in patients with malignant tumors based on their inflammatory continuous prognostic score (18–20). A previous study has reported that CRP to Lymphocyte Ratio (CLR) and CRP might be better than LYM alone in assessing patients with severe COVID-19 because CLR is a highly sensitive measure to evaluate the severity of COVID-19 in the early phase (21). However, the sample size of that study was small and the association between CLR and patients’ outcomes was not explored. In this retrospective study, it is our aim to evaluate the effectiveness and clinical applicability of CLR in predicting clinical outcomes of SARS-CoV-2 BA.2.2 patients during their admission.

## 2. Materials and methods

### 2.1. Study population and design

**Figure 1:**
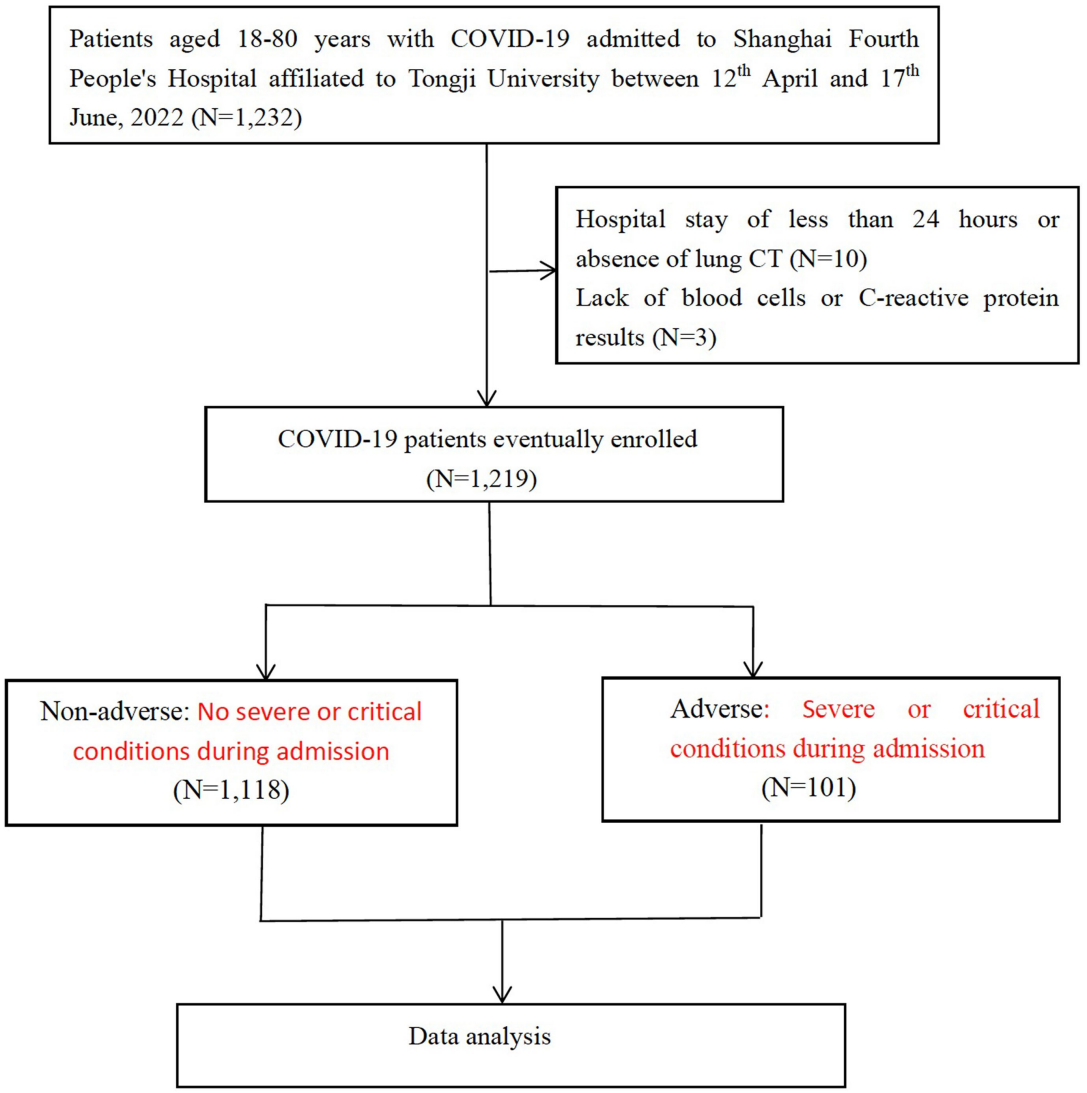
Flow chart of patient recruitment, clinical screening, and evaluation.

This study is a single-center, retrospective, observational study on confirmed COVID-19 patients who were admitted to Shanghai Fourth People’s Hospital affiliated to Tongji University between 12th April, 2022 and 17th June, 2022. The diagnosis of COVID-19 was confirmed using PCR tests. Patients with positive SARS-CoV-2 BA.2.2 PCR tests were included in the present study. Patients were excluded if they met any of the following criteria: (1) < 18, or > 80 years; (2) missing blood cell counts or C-reactive protein results; (3) COVID-19 genotyping was impossible because of hospital stay less than 24 h or absence of CT to scan the lungs (Figure 1). The present study was approved by the human ethics committee of Shanghai Fourth People’s Hospital and written informed consent was waived due to the nature of being a retrospective study.

### 2.2. Data collection

Data were collected from electronic medical records of hospitalized patients, including demographic data, such as age, gender, and concomitant conditions like hypertension, coronary heart disease, atrial fibrillation, diabetes, stroke, dementia, as well as parkinsonism, therapies like oxygen support, antiviral therapy, and use of corticosteroids, laboratory data, such as red blood cell counts, white blood cell counts, neutrophil counts, monocyte counts, lymphocyte counts, platelet counts of the peripheral blood, levels of hemoglobin in the plasma and levels of serum CRP, alanine transaminase, aspartate aminotransferase, creatinine, lactate dehydrogenase, troponin-I, d-dimer, procalcitonin, neutrophil lymphocyte ratio (NLR), platelet lymphocyte ratio (PLR), monocyte lymphocyte ratio (MLR), and CLR, which were obtained within 24 h of admission. All patients were followed up during their admission period and their outcomes, such as death or being discharged were recorded. Some patients were admitted twice during the study period due to the repeated procedures of admission, and the worse outcome was used for final analysis. The primary outcome in this study was severe or critical conditions during admission.

The COVID-19 clinical genotyping criteria complied with those from China’s official clinical guidelines. Adults can be diagnosed with severe COVID-19 if they met any of the following criteria: (1) shortness of breath, breathing rate greater than or equal to 30 times/min; (2) oxygen saturation is less than or equal to 93% during air inhalation at rest; (3) the ratio of arterial partial pressure of oxygen (PaO_2_) to oxygen concentration (FiO_2_) < 300 mmHg; PaO_2_/FiO_2_ was adjusted using a formula which is for correction at high altitudes (eg above 1,000 m): PaO_2_/FiO_2_ × [760/atmospheric pressure (mmHg)]. (4) patients whose conditions progressively deteriorated and lesions on lung imaging significantly expanded by >50% within 24–48 h. Critical COVID-19 can be diagnosed if patients presented with one of the following conditions: (1) respiratory failure and the need for mechanical ventilation; (2) the appearance of shock; and (3) intensive care and treatment initiated due to other complications, such as other organ failure.

### 2.3. Statistical analysis

Continuous variables were expressed in the form of median and interquartile range (IQR) or mean ± SD, whereas categorical variables were expressed in the form of absolute numbers and frequencies (%). Results were compared between groups using either independent sample *t*-tests, or Mann–Whitney U-tests, or Chi-square or Fischer’s exact tests as required. Receiver operating characteristic (ROC) curve was used to assess the severity of COVID-19. Youden index was used to evaluate the authenticity of screening tests. The value corresponding to the maximum Youden index is taken as the cut-off value. Models of multivariate logistic regression were built to calculate the odds ratios (ORs), adjusted ORs, and their corresponding 95% CIs for correlations between CLR and clinical outcomes. Confounding factors for these models were selected based on published literatures and clinical judgment, focusing on variables that might confound the relationship between CLR and clinical outcomes. Models were first adjusted for gender and age (model A), and then adjusted for other confounders, such as hypertension, coronary artery disease, atrial fibrillation, diabetes, stroke, dementia, and parkinsonism (model B). In model C, age, sex, hypertension, diabetes, coronary artery disease, atrial fibrillation, history of stroke, dementia, parkinsonism, oxygen support, antiviral therapy, and use of corticosteroid were adjusted. Statistical analyses were conducted using the Statistical Package for the Social Sciences (SPSS version 22). Values of *p* < 0.05 were considered statistically significant.

## 3. Results

### 3.1. Participant characteristics

A total of 1,232 COVID-19 patients met the inclusion criteria and were included in the present study. Three patients who lacked blood cell counts or CRP results, 10 who were unable to complete COVID-19 genotyping due to the length of hospital stay less than 24 h or lack of pulmonary CT examination were excluded. In the end, 1,219 patients were enrolled. The selection process was shown in Figure 1, and baseline characteristics of included patients were shown in Table 1.

**Table 1:** Baseline characteristics of patients with adverse and non-adverse outcomes.

Variables	Total patients	Non-adverse	Adverse	*p* value
Patients, *n* (%)	1,219 (100%)	1,118 (91.7%)	101 (8.3%)	
Sex, *n* (%)				0.011
Female	619 (50.8%)	580 (51.9%)	39 (38.6%)	
Male	600 (49.2%)	538 (48.1%)	62 (61.4%)	
Age, median (IQR), years	68 (60,73)	67 (59,73)	71 (66,76)	<0.001
Comorbidities, *n* (%)				
Hypertension	429 (35.2%)	382 (34.2%)	47 (46.5%)	0.013
Diabetes	228 (18.7%)	203 (18.2%)	25 (24.8%)	0.104
Cardiovascular disease	17 (1.4%)	14 (1.3%)	3 (3%)	0.161
Atrial fibrillation	26 (2.1%)	23 (2.1%)	3 (3%)	0.470
History of stroke	115 (9.4%)	86 (7.7%)	29 (28.7%)	<0.001
Dementia	28 (2.3%)	17 (1.5%)	11 (10.9%)	<0.001
Parkinsonism	16 (1.3%)	12 (1.1%)	4 (4%)	0.037
Laboratory testing				
CRP, mg/L	5.55 (2.06,15.9)	4.85 (1.86,12.86)	49.25 (21.19,162.06)	<0.001
White blood cell count, ×10^9/L	5.27 (4.23,6.78)	5.20 (4.21,6.66)	6.64 (5.01,9.37)	<0.001
Neutrophil count, ×10^9/L	3.18 (2.32, 4.47)	3.08 (2.27,4.30)	4.83 (3.57,7.84)	<0.001
Lymphocyte count, ×10^9/L	1.39 (0.96,1.87)	1.43 (1.01,1.89)	0.82 (0.52,1.40)	<0.001
Monocyte count, ×10^9/L	0.42 (0.32,0.56)	0.42 (0.33,0.56)	0.38 (0.28,0.57)	0.100
Platelet count, ×10^9/L	185 (145, 231)	186.50 (146.75,233)	172 (130, 216.50)	0.023
Red blood cell count, ×10^12/L	4.32 (3.97, 4.70)	4.34 (3.99, 4.70)	4.17 (3.57,4.70)	0.005
Hemoglobin, g/L	129 (119,140)	130 (120,140)	125 (104.50,139)	0.005
Alanine transaminase, U/L	18.85 (13.33,28.54)	18.63 (13.29,27.74)	22.56 (13.80,37.29)	0.035
Aspartate aminotransferase, U/L	23.08 (18.42,30.04)	22.62 (18.22,29.12)	35.30 (23.01,56.25)	<0.001
Creatinine,μmoI/L	56.80 (47.80,69.90)	57.05 (48.20,69.50)	53.90 (41.40,74.30)	0.092
Lactate dehydrogenase, U/L	182.78 (146.25,216.69)	181.04 (145.37,210.76)	228.69 (154.87,296.35)	<0.001
Troponin-I,μg/L	0.01 (0.00,0.02)	0.01 (0.00,0.02)	0.02 (0.01,0.03)	<0.001
D-dimer, mg/L	0.40 (0.26,0.76)	0.37 (0.25,0.63)	1.34 (0.77,2.32)	<0.001
Procalcitonin, μg/L	0.02 (0.02,0.03)	0.02 (0.02,0.02)	0.19 (0.03,0.71)	<0.001
NLR	2.33 (1.46,3.72)	2.20 (1.42,3.46)	5.63 (2.85,12.89)	<0.001
PLR	133.07 (99.15,189.06)	130.12 (97.26,184.49)	194.44 (121.80,314.03)	<0.001
MLR	0.29 (0.21,0.48)	0.28 (0.21,0.46)	0.45 (0.27,0.80)	<0.001
CLR, mg/10^9	4.25 (1.35,13.87)	3.70 (1.22,10.42)	59.60 (15.89,242.74)	<0.001
Therapies				
Oxygen support				<0.001
No oxygen support	850 (69.7%)	835 (74.7%)	15 (14.9%)	
Ordinary oxygen support	319 (26.2%)	278 (24.9%)	41 (40.6%)	
Non-normal oxygen support	50 (4.1%)	5 (0.4%)	45 (44.5%)	
Antiviral therapy	877 (71.9%)	803 (71.8%)	74 (73.3%)	0.757
Use of corticosteroid	63 (5.2%)	24 (2.1%)	39 (38.6%)	*p* < 0.01

NLR, neutrophil lymphocyte ratio; PLR, platelet lymphocyte ratio; MLR, monocyte lymphocyte ratio; and CLR, C-reactive protein lymphocyte ratio. Ordinary oxygen support: normal nasal tube oxygen; Non-normal oxygen support: high flow oxygen or ventilator-assisted oxygen; Antiviral therapy: the antiviral drug is Paxlovid.

In the present study, 101 patients (8.3%) had adverse outcomes. Percentages of male and female patients in the adverse outcome group were comparable to those in the non-adverse outcome group, but there were significantly more males than females in the adverse outcome group, and the average age of patients in the adverse outcome group was larger than that in the non-adverse outcome group. These differences were statistically significant (*p* < 0.05). Regarding concomitant comorbidities, percentages of hypertension, dementia, stroke, and Parkinson’s disease in the adverse outcome group were significantly larger than those in the non-adverse outcome group (*p* < 0.05). In terms of treatment, oxygen support and use of corticosteroids showed statistical difference, while antiviral therapy showed no significant difference between adverse and non-adverse outcomes. Levels of CRP, leukocyte counts, neutrophil counts, alanine transaminase, aspartate aminotransferase, lactate dehydrogenase, troponin-I, d-dimer, and procalcitonin were significantly higher in patients with adverse outcomes than those without adverse outcomes. Lymphocyte counts, monocyte counts and platelet count, red blood cell counts, and levels of hemoglobin and creatinine were relatively lower in patients with adverse outcomes than those without adverse outcomes. Apart from the mononuclear cell count and creatinine, other differences were also statistically significant (*p* < 0.05). Furthermore, patients with adverse outcomes had the largest increase in CRP and the most significant decrease in lymphocyte counts.

### 3.2. Comparison of the area under ROC curve of lymphocyte counts, CRP, and CLR

**Figure 2:**
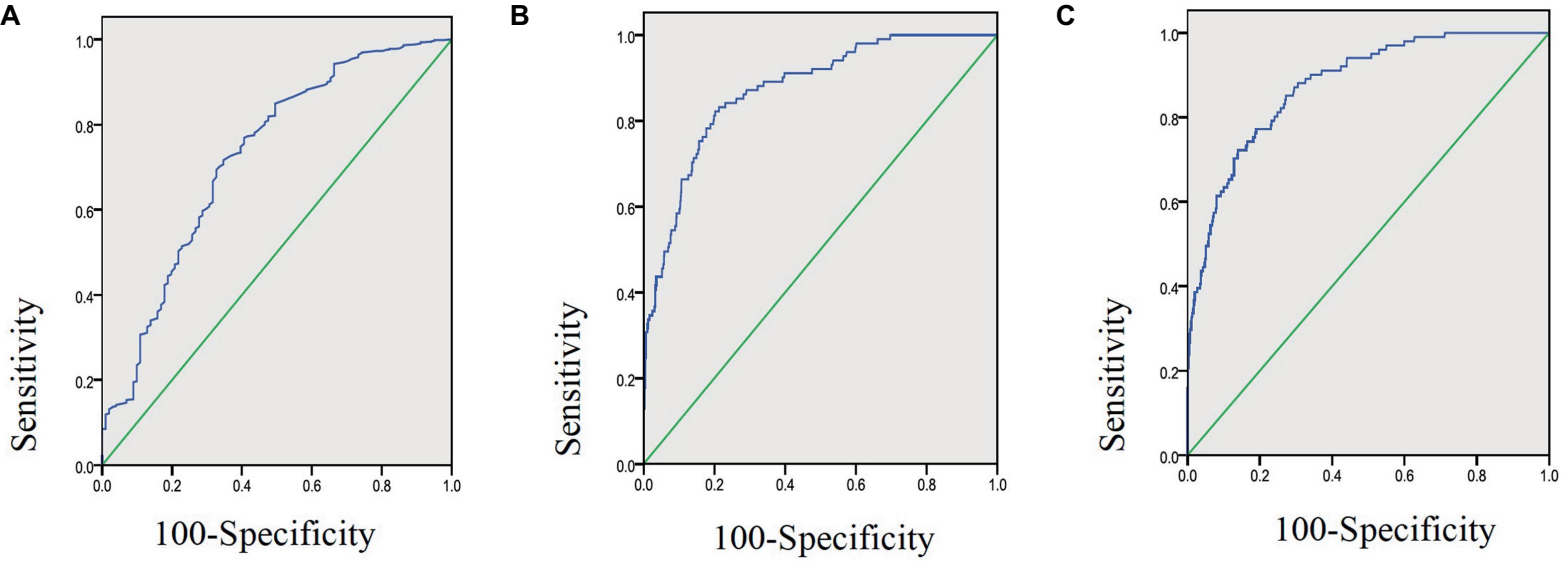
Area under ROC curve of lymphocyte count **(A)**, CRP **(B)**, and CLR **(C)** in 1,219 COVID-19 patients aged 18–80 years.

As shown in Table 2, the effectiveness of CRP, LYM, and CLR in predicting adverse outcomes was compared, and the areas under the receiver operating characteristic curves (ROC) were 0.872, 0.724, and 0.877, respectively. The ROC of CLR was the largest (Figure 2), with a cut-off value of 21.25, a sensitivity of 72.3% and a specificity of 86%.

**Table 2:** Characteristics of ROC curves in COVID-19 patients.

	ACU (95%CI)	SE	Youden index	Cut-off	Sensitivity	Specificity	*p* value
CRP	0.872 (0.838–0.907)	0.018	0.62	15.75	82.2%	79.8%	<0.001
LYM	0.724 (0.668–0.780)	0.029	0.369	1.065	71.6%	65.3%	<0.001
CLR	0.877 (0.843–0.910)	0.017	0.583	21.25	72.3%	86%	<0.001

SE, standard error; CRP, C-reactive protein; LYM, lymphocyte count; and CLR, the ratio of C-reactive protein to Lymphocyte count.

### 3.3. Patient characteristics associated with the cut-off value of CLR

In Table 3, included patients were divided into two groups according to the cut-off value of CLR (21.25). There were 229 patients whose ROC was greater than the cut-off value (high group) and 990 smaller than the cut-off value (low group), accounting for 18.8 and 81.2%, respectively. The proportion of male patients in the high group was significantly larger than that in the low group, and similar phenomenon was observed in the average age (*p* < 0.05). Regarding concomitant comorbidities, there were significant differences in diabetes, stroke, and Parkinson’s disease (*p* < 0.05). In terms of treatment, oxygen support and use of corticosteroids showed statistical difference, while antiviral therapy showed no significant difference between adverse and non-adverse outcome groups (*p* < 0.05). In laboratory tests, significant differences were observed in CRP, white blood cell counts, lymphocyte counts, monocyte counts, platelet counts, red blood cell counts, and levels of hemoglobin, aspartate aminotransferase, creatinine, lactate dehydrogenase, troponin-I, d-dimer, procalcitonin, NLR, PLR, MLR, and CLR (*p* < 0.05).

**Table 3:** Baseline characteristics of patients stratified by CLR.

Variables	Total patients	CLR < 21.25	CLR ≥ 21.25	*p* value
Patients, *n* (*%*)	1,219	990 (81.2%)	229 (18.8%)	
Sex, *n* (*%*)				<0.001
Female	619 (50.8%)	558 (56.4%)	61 (26.6%)	
Male	600 (49.2%)	432 (43.6%)	168 (73.4%)	
Age, median (IQR), years	68 (60,73)	67 (59,73)	69 (62,75)	0.002
Comorbidities, *n* (*%*)				
Hypertension	429 (35.2%)	342 (34.5%)	87 (38%)	0.325
Diabetes	228 (18.7%)	168 (17%)	60 (26.2%)	0.001
Cardiovascular disease	17 (1.4%)	15 (1.5%)	2 (0.9%)	0.754
Atrial fibrillation	26 (2.1%)	20 (2%)	6 (2.6%)	0.61
History of stroke	115 (9.4%)	73 (7.4%)	42 (18.3%)	<0.001
Dementia	28 (2.3%)	20 (2.0%)	8 (3.5%)	0.18
Parkinsonism	16 (1.3%)	9 (0.9%)	7 (3.1%)	0.019
Laboratory testing				
CRP, mg/L	5.55 (2.06,15.9)	4.00 (1.57,8.56)	57.09 (33.91,113.58)	<0.001
White blood cell count, ×10^9/L	5.27 (4.23,6.78)	5.14 (4.19,6.36)	6.61 (4.51,8.99)	<0.001
Neutrophil count, ×10^9/L	3.18 (2.32,4.47)	2.99 (2.18,4.04)	4.88 (3.27,7.36)	<0.001
Lymphocyte count, ×10^9/L	1.39 (0.96,1.87)	1.50 (1.09,1.93)	0.88 (0.61,1.27)	<0.001
Monocyte count, ×10^9/L	0.42 (0.32,0.56)	0.41 (0.32,0.54)	0.47 (0.33,0.64)	0.003
Platelet count, ×10^9/L	185 (145, 231)	187 (148,232.25)	172 (133.5,230.5)	0.04
Red blood cell count, ×10^12/L	4.32 (3.97,4.70)	4.37 (4.04,4.73)	4.15 (3.57,4.62)	<0.001
Hemoglobin, g/L	129 (119,140)	131 (121,140.25)	122 (106,137.50)	<0.001
Alanine transaminase, U/L	18.85 (13.33,28.54)	18.73 (13.36,27.68)	19.46 (12.80,32.05)	0.417
Aspartate aminotransferase, U/L	23.08 (18.42,30.04)	22.50 (18.25,28.89)	26.66 (19.22,43.00)	<0.001
Creatinine,μmoI/L	56.80 (47.80,69.90)	56.35 (47.90,68.08)	60.80 (46.60,78.40)	0.029
Lactate dehydrogenase, U/L	182.78 (146.25,216.69)	179.50 (144.30,206.00)	208.50 (154.70,260.50)	<0.001
Troponin-I,μg/L	0.01 (0.00,0.02)	0.01 (0.00,0.01)	0.01 (0.01,0.02)	<0.001
D-dimer, mg/L	0.40 (0.26,0.76)	0.35 (0.24,0.57)	0.91 (0.47,1.64)	<0.001
Procalcitonin, μg/L	0.02 (0.02,0.03)	0.03 (0.02,2.53)	0.09 (0.02,0.36)	<0.001
NLR	2.33 (1.46,3.72)	2.01 (1.33,3.00)	5.27 (3.29,9.42)	<0.001
PLR	133.07 (99.15,189.06)	124.24 (93.53,172.71)	195.05 (129.27,307,14)	<0.001
MLR	0.29 (0.21,0.48)	0.34 (0.27,1.83)	0.67 (0.51,9.40)	<0.001
CLR, mg/10^9	4.25 (1.35,13.87)	2.79 (1.09,7.13)	59.60 (32.94,126.29)	<0.001
Therapies				
Oxygen support				<0.001
No oxygen support	850 (69.7%)	743 (75.1%)	107 (46.7%)	
Ordinary oxygen support	319 (26.2%)	232 (23.4%)	87 (38.0%)	
Non-normal oxygen support	50 (4.1%)	15 (1.5%)	35 (15.3%)	
Antiviral therapy	877 (71.9%)	721 (72.8%)	156 (68.1%)	0.153
Use of corticosteroid	63 (5.2%)	27 (2.7%)	36 (15.7%)	<0.001

NLR, neutrophil lymphocyte ratio; PLR, platelet lymphocyte ratio; MLR, monocyte lymphocyte ratio; CLR, C-reactive protein lymphocyte ratio Ordinary oxygen support: Normal nasal tube oxygen, Non-normal oxygen support: High flow oxygen or ventilator-assisted oxygen. Antiviral therapy: the antiviral drug is Paxlovid.

### 3.4. Association of CLR with adverse clinical outcomes

In Table 4, clinical outcomes were compared between the high and the low groups. It was found that the percentages of adverse outcomes were 31.9 and 2.8%, respectively. The proportions of severely and critically ill patients were 27.1 and 2.3%. These differences between the two groups were significant (*p* < 0.001).

**Table 4:** Association between CLR and clinical outcomes.

	CLR < 21.25	CLR ≥ 21.25	*p* value	Chi-square value
	(*N* = 990)	(*N* = 229)		
Adverse outcomes	28 (2.8%)	73 (31.9%)	*p* < 0.001	206.532
Non-adverse	962 (97.2%)	156 (68.1%)		

CLR: the ratio of C-reactive protein to Lymphocyte count.

### 3.5. Correlation between CLR and the risk of adverse outcomes

In Table 5, the logistic regression model was used to evaluate the correlation between CLR (below/above CLR cut-off) and the risks of adverse clinical outcomes (severe/critical) in COVID-19 patients. Model A: age, sex-adjusted; Model B: multivariate-adjusted, including age, sex, hypertension, atrial fibrillation, history of stroke, dementia, and Parkinsonism. Model C: Including age, sex, hypertension, diabetes, coronary artery disease, atrial fibrillation, history of stroke, dementia, parkinsonism, oxygen support, antiviral therapy, and use of corticosteroids. From these four models, it could be seen that CLR with a cut-off value of 21.25 was a potential predictor for adverse outcomes. Regarding adverse outcomes, the unadjusted odds ratio (OR) was 16.08, and the adjusted OR (aOR) after adjusting age and sex (Model A) was 17.09, and the aORs of the multi-factor adjusted models (model B and model C) were 17.04 and 12.29, suggesting that a large CLR was significantly associated with a high risk of poor prognosis.

**Table 5:** Correlation between CLR and the risks of adverse clinical outcomes.

	Adverse clinical outcomes		
	^a^OR	95%CI	*p* value
Unadjusted	16.08	10.08–25.66	<0.001
Age, sex-adjusted (Model A)	17.09^a^	10.33–28.28	<0.001
Multivariate 1-adjusted (Model B)	17.04^a^	10.07–28.83	<0.001
Multivariate 2-adjusted (Model C)	12.29^a^	6.24–24.20	<0.001

Model A: adjusted for age and gender. Model B: adjusted for multivariable, including age, sex, hypertension, diabetes, coronary artery disease, atrial fibrillation, history of stroke, dementia, and parkinsonism. Model C: including age, sex, hypertension, diabetes, coronary artery disease, atrial fibrillation, history of stroke, dementia, parkinsonism, oxygen support, antiviral therapy, and use of corticosteroid. ^a^OR represents adjusted OR valule.

## 4. Discussion

2019 corona virus disease has been ongoing for 3 years. Although vaccination among the general population has significantly decreased disease mortality and even the incidence in various regions (22), there are still challenges in confronting the uncertainties introduced by recently identified variants of the COVID-19 virus. It is the experience of multiple clinical centers that the systemic inflammatory panel could reliably predict the exacerbation of this disease 20 days before its occurrence (23, 24). If previously reported approaches can be applied to patients infected by Omicron variants, priorities of receiving specialized treatments can be allocated to those whose conditions are deteriorating. The goal of the present study was to explore the characteristics of Omicron variant inflicted patients and predict their outcomes based on previous knowledge and experience. The baseline data of the present study (Table 1) showed that there were 101 patients (8.3%) with poor prognosis (including severe illness, critical illness) among COVID-19 patients aged between 18 and 80. Although the rate of critical illness was not high in the entire population of COVID-19 patients, it is important to note that patients with adverse outcomes were more likely to be males, older elderlies and those who have multiple comorbidities.

It has been reported that systemic inflammation due to COVID-19 infection leads to immune suppression and apoptosis of lymphocytes (25). This might be the result of direct cytotoxicity of this virus to lymphocytes as this virus was found present in circulating lymphocytes (19, 26). However, the level of CRP has been shown to rise earlier than either lymphopenia or neutrophilia (27). CRP, a super-early reactive protein, is considered to be a hallmark of response to inflammatory cytokines associated with monocyte or macrophage activation, and its expression is increased in inflammatory conditions (21). In certain cases, CRP can activate the complement system, further augmenting the release of inflammatory cytokines, exacerbating tissue damage (28). Therefore, the significantly elevated CRP may reflect the severity of inflammation, whereas lymphopenia is associated with suppressed immune function and adverse outcomes of COVID-19 patients, and CLR may be more sensitive in capturing the early part of the inflammatory cascade than other biomarkers as previously reported (19, 21, 26–28). Our study on Omicron BA.2.2 further verified that CLR was more superior (Table 2) to CRP or Lymphocyte counts alone, evidenced by its cutoff value of 21.25 demonstrating a sensitivity of 72.30% and a specificity of 86%. The area under the ROC (AUC) of CLR was 0.877 (95%CI: 0.843–0.910), and it was the largest compared with that of CRP or Lymphocyte counts alone (Figure 2). Our results showed that CRP had a predictive sensitivity of 82.2% for adverse prognosis after Omicron infection. Therefore, we suggest that CRP should be used to screen severe COVID-19 patients and CLR should be used to predict the prognosis of patients at the early stage. These two indicators are easy and soon to obtain from every patient, which will facilitate early patient triage and save limited medical resources.

Consistent with what was previously reported in Wuhan, China (21), the present study found significant differences in gender and age between the two groups of patients with the CLR above 21.25 group having significantly more males and older patients than the CLR below 21.25 group. In addition, this is the first report on the association between neurological comorbidities and the prognosis of COVID-19 patients evidenced by the significant difference in stroke and Parkinson’s disease between these two groups. Furthermore, the association between CLR and adverse outcomes found in the present study and the regression analysis suggests that CLR is an independent risk factor for poor prognosis of Omicron BA.2.2 patients at the cut-off value of 21.25. Our findings imply that CLR is a more sensitive biomarker than CRP or the lymphocyte count alone in predicting patients’ prognosis after COVID-19 infection. This might be applied to other infectious conditions or inflammatory conditions.

### 4.1. Limitations

A number of limitations were present in our study. First, it is a single center, retrospective study. Therefore, selection bias and other limitations may confine the extrapolation of our conclusion. For example, only some commonly observed confounders were included in the multivariate regression analyses. Second, the present study aimed to focus on findings at admission as predictive markers for adverse outcomes, hence our multivariate regression analyses did not include the type and timing of treatments as variables, which may impact clinical outcomes of COVID-19 patients. In a meta-analysis study, it was found from 44 studies including 20,197 patients that corticosteroids were beneficial for short-term mortality and for mechanical ventilation (29). Third, CLR was only assessed at admission to hospital. The impact of dynamic changes of CRP, lymphocyte counts on clinical outcomes was not evaluated. Additionally, lymphopenia was shown in the full blood test, but which subtypes of lymphocytes were decreased were not known. Further investigations on this may provide deeper insight into disease progression mechanisms and estimation of clinical outcomes of COVID-19 patients. In spite of these limitations, our conclusion was drawn from a relatively large population and the findings were consistent with those of previous studies (21, 26, 27). Furthermore, we have analyzed more clinical and biochemical parameters in our regression model. There are not many studies on this new strain of Omicron. Therefore, this study has its own innovative characters.

## 5. Conclusion

The present study found that the overall rate of adverse outcomes (severely or critically ill) after Omicron BA.2.2 infection in adults aged 18–80 years is not high. CRP increased the most and lymphocyte count decreased the most within 24 h after admission. CLR is better than CRP or LYM alone in predicting poor prognosis. The cutoff value of CLR 21.25 is an independent predictor of poor prognosis of Omicron BA.2.2 inflicted patients. Early application of this CLR cut-off value to predict poor prognosis is conducive to patient triage and allocation of medical resources.

## Data availability statement

The original contributions presented in the study are included in the article/supplementary material, further inquiries can be directed to the corresponding author.

## Ethics statement

This study involving human participants were reviewed and approved by the Human Ethics Committee of Shanghai Fourth People’s Hospital.

## Author contributions

YB and YW conceived this study. BX and YW collected and analyzed data. YH and JX helped with data analysis. ZY helped with data collection. BX and HL wrote the draft. YB revised the manuscript. All authors contributed to the article and approved the submitted version.

## Conflict of interest

The authors declare that the research was conducted in the absence of any commercial or financial relationships that could be construed as a potential conflict of interest.

## Publisher’s note

All claims expressed in this article are solely those of the authors and do not necessarily represent those of their affiliated organizations, or those of the publisher, the editors and the reviewers. Any product that may be evaluated in this article, or claim that may be made by its manufacturer, is not guaranteed or endorsed by the publisher.
